# Joint association of mammographic density adjusted for age and body mass index and polygenic risk score with breast cancer risk

**DOI:** 10.1186/s13058-019-1138-8

**Published:** 2019-05-22

**Authors:** Celine M. Vachon, Christopher G. Scott, Rulla M. Tamimi, Deborah J. Thompson, Peter A. Fasching, Jennifer Stone, Melissa C. Southey, Stacey Winham, Sara Lindström, Jenna Lilyquist, Graham G. Giles, Roger L. Milne, Robert J. MacInnis, Laura Baglietto, Jingmei Li, Kamila Czene, Manjeet K. Bolla, Qin Wang, Joe Dennis, Lothar Haeberle, Mikael Eriksson, Peter Kraft, Robert Luben, Nick Wareham, Janet E. Olson, Aaron Norman, Eric C. Polley, Gertraud Maskarinec, Loic Le Marchand, Christopher A. Haiman, John L. Hopper, Fergus J. Couch, Douglas F. Easton, Per Hall, Nilanjan Chatterjee, Montse Garcia-Closas

**Affiliations:** 1 Division of Epidemiology, Department of Health Sciences Research, Mayo Clinic, Rochester, 55905 MN USA; 2 Division of Biomedical Statistics and Informatics, Mayo Clinic, Rochester, 55905 MN USA; 3 Channing Division of Network Medicine, Department of Medicine, Brigham and Women’s Hospital, Harvard Medical School, Boston, 02115 MA USA; 4 Department of Epidemiology, Harvard TH Chan School of Public Health, Boston, MA 02115 USA; 5 Program in Genetic Epidemiology and Statistical Genetics, Harvard TH Chan School of Public Health, Boston, MA 02115 USA; 6 Centre for Cancer Genetic Epidemiology, Department of Public Health and Primary Care, University of Cambridge, Cambridge, CB1 8RN UK; 7 Department of Gynecology and Obstetrics, Comprehensive Cancer Center Erlangen Nuremberg, University Hospital Erlangen, Friedrich-Alexander-University Erlangen-Nuremberg, 91054 Erlangen, Germany; 8 Department of Medicine, Division of Hematology and Oncology, University of California at Los Angeles, David Geffen School of Medicine, Los Angeles, CA 90095 USA; 9 The Curtin UWA Centre for Genetic Origins of Health and Disease, Curtin University and University of Western Australia, Perth, Western Australia 6009 Australia; 10 Centre for Epidemiology and Biostatistics, Melbourne School of Population and Global Health, The University of Melbourne, Melbourne, Victoria 3010 Australia; 11 Precision Medicine, School of Clinical Sciences at Monash Health, Monash University, Clayton, Victoria 3168 Australia; 12 Department of Clinical Pathology, The University of Melbourne, Melbourne, Victoria 3010 Australia; 13 Department of Epidemiology, University of Washington School of Public Health, Seattle, WA 98195 USA; 14 Public Health Sciences Division, Fred Hutchinson Cancer Research Center, Seattle, WA 98109 USA; 15 Cancer Epidemiology and Intelligence Division, Cancer Council Victoria, Melbourne, Victoria 3004 Australia; 16 Department of Epidemiology and Preventive Medicine, Monash University, Melbourne, Victoria Australia; 17 Human Genetics, Genome Institute of Singapore, Singapore, Singapore; 18 Department of Medical Epidemiology and Biostatistics, Karolinska Institutet, 171 65 Stockholm, Sweden; 19 Department of Public Health and Primary Care, University of Cambridge, Cambridge, CB1 8RN UK; 20 Medical Research Council (MRC) Epidemiology Unit, Institute of Metabolic Science, University of Cambridge, Cambridge, CB1 8RN UK; 21 Epidemiology Program, University of Hawaii Cancer Center, Honolulu, 96813 HI USA; 22 Department of Preventive Medicine, Keck School of Medicine, University of Southern California, Los Angeles, CA 90033 USA; 23 Department of Laboratory Medicine and Pathology, Mayo Clinic, Rochester, MN 55905 USA; 24 Centre for Cancer Genetic Epidemiology, Department of Oncology, University of Cambridge, Cambridge, CB1 8RN UK; 25 Department of Oncology, South General Hospital, 118 83 Stockholm, Sweden; 26 Division of Cancer Epidemiology and Genetics, National Cancer Institute, Bethesda, MD 20892 USA; 27 Department of Biostatistics, Bloomberg School of Public Health, John Hopkins University, Baltimore, 21218 MD USA; 28 Department of Oncology, School of Medicine, John Hopkins University, Baltimore, 21218 MD USA; 29 Division of Cancer Epidemiology and Genetics, National Cancer Institute, Rockville, MD 20850 USA; 30 Department of Clinical and Experimental Medicine, University of Pisa, Pisa, Italy

**Keywords:** Breast density, Breast cancer risk, Polygenic risk score, Genetic variation, Risk models

## Abstract

**Background:**

Mammographic breast density, adjusted for age and body mass index, and a polygenic risk score (PRS), comprised of common genetic variation, are both strong risk factors for breast cancer and increase discrimination of risk models. Understanding their joint contribution will be important to more accurately predict risk.

**Methods:**

Using 3628 breast cancer cases and 5126 controls of European ancestry from eight case-control studies, we evaluated joint associations of a 77-single nucleotide polymorphism (SNP) PRS and quantitative mammographic density measures with breast cancer. Mammographic percent density and absolute dense area were evaluated using thresholding software and examined as residuals after adjusting for age, 1/BMI, and study. PRS and adjusted density phenotypes were modeled both continuously (per 1 standard deviation, SD) and categorically. We fit logistic regression models and tested the null hypothesis of multiplicative joint associations for PRS and adjusted density measures using likelihood ratio and global and tail-based goodness of fit tests within the subset of six cohort or population-based studies.

**Results:**

Adjusted percent density (odds ratio (OR) = 1.45 per SD, 95% CI 1.38–1.52), adjusted absolute dense area (OR = 1.34 per SD, 95% CI 1.28–1.41), and the 77-SNP PRS (OR = 1.52 per SD, 95% CI 1.45–1.59) were associated with breast cancer risk. There was no evidence of interaction of the PRS with adjusted percent density or dense area on risk of breast cancer by either the likelihood ratio (*P* > 0.21) or goodness of fit tests (*P* > 0.09), whether assessed continuously or categorically. The joint association (OR) was 2.60 in the highest categories of adjusted PD and PRS and 0.34 in the lowest categories, relative to women in the second density quartile and middle PRS quintile.

**Conclusions:**

The combined associations of the 77-SNP PRS and adjusted density measures are generally well described by multiplicative models, and both risk factors provide independent information on breast cancer risk.

**Electronic supplementary material:**

The online version of this article (10.1186/s13058-019-1138-8) contains supplementary material, which is available to authorized users.

## Introduction

Large consortia have identified multiple common genetic susceptibility markers associated with risk of breast cancer [1–4]. Although each single nucleotide polymorphism (SNP) is associated with a small increase in risk, their combined effects are more substantial [5, 6]. Effects of multiple SNPs have been shown to combine multiplicatively, such that the combined effects can be efficiently summarized as polygenic risk scores (PRS) [2, 6]. Based on a 77-SNP PRS, women in the top 10% of the risk distribution have been estimated to have a two-fold risk of breast cancer, relative to those of median PRS, and this risk increases to three-fold for women in the top 1% [6]. In addition, several studies have shown that the PRS is a strong risk factor for young women [7], those with family history [8], *BRCA1* and *BRCA2* mutation carriers [7, 9–11], and for women with contralateral breast cancer [12]. Use of the PRS has also been shown to increase the discrimination of risk models [4, 13–16]. The PRS therefore has the potential to add information to the established risk factors for breast cancer and improve individualized risk prediction [17].

Understanding joint associations of the PRS with other risk factors is important for accurate risk prediction [6, 17–19]. In the most comprehensive study to examine the joint association of a breast cancer PRS and environmental factors (defined as reproductive, anthropometric, lifestyle factors and exogenous hormones) on risk, based on up to 28,241 and 30,445 controls, most associations were consistent with independent (i.e., multiplicative) associations [17]. This implies that the higher a woman’s genetic risk, the greater the absolute risk associated with environmental risk factors [17, 20].

Mammographic density adjusted for age and BMI is one of the strongest breast cancer risk factors [21, 22] but few studies have examined the joint relationship of mammographic density measures and PRS on breast cancer risk. We previously estimated the contribution of the American College of Radiology (ACR) Breast Imaging Reporting and Data System (BI-RADS) four category density measure and a 76-SNP PRS with breast cancer risk using three studies [19]. We found the PRS and BI-RADS density were independent breast cancer risk factors (with no evidence that their joint association deviated from multiplicative) and that the PRS improved discrimination of the Breast Cancer Surveillance Consortium (BCSC) risk model [11, 19]. However, these prior studies lacked the precision of a quantitative mammographic density measure, did not examine absolute dense area, and had limited power for evaluating interactions. A continuous measure could provide better risk discrimination than a categorical measure such as BI-RADS (which has only four categories) [22] and particularly in the tails of the distributions, where clinical implications will be the greatest; the highest risk women could be offered more intensive screening or interventions while women with lowest risk could have reduced or less frequent screening [18].

Here, we evaluate the joint associations on breast cancer risk of a 77-SNP PRS for breast cancer and quantitative mammographic density measures, including percent density and absolute dense area, adjusted for age and BMI, using data from eight studies in the Breast Cancer Association Consortium (BCAC) [1, 23–27].

## Methods

### Subjects

The study sample consisted of 3628 cases and 5126 controls of European ancestry from eight studies in BCAC; of these, six studies were population-based, contributing 2439 cases and 3895 controls, and the others were clinic based. Each study had available genotyping information on the 77 SNPs included in the PRS, mammographic density and other breast cancer risk factors. Each study obtained informed consent and had relevant ethics and institutional approvals. A summary of study design, sample sizes, and mammographic and genotyping characteristics is given in Additional file 1: Table S1*.*

### Mammographic density measures

All mammographic density measurements were performed on digitized analogue films using either the Cumulus [28] or Madena [29] programs (Additional file 1: Table S1) which apply a thresholding technique to measure total area of the breast and absolute dense area, from which percent dense area and absolute non-dense area are derived. Absolute dense area values were converted to square centimeters according to the pixel size used in the digitization. Measurements were conducted by observers blind to genotype, case status, and breast cancer risk factor data. For cases, mammograms prior to diagnosis or, when this was not possible, those from the contralateral breast taken at the time of diagnosis were used (Additional file 1: Table S1). The mammographic density measurements were made for both craniocaudal (CC) and mediolateral oblique (MLO) views, which have consistently been shown to be highly correlated (range 0.87–0.90) [30]. All studies have previously contributed to genetic analyses of mammographic density, and similar associations were found across studies [31–34].

### Genotyping

The 77 SNPs used to compute the PRS [6] were genotyped for the eight studies either as part of a GWAS (Illumina, Human Hap550) [34] or on a custom Illumina iSelect genotyping array comprising 211,155 SNPs (iCOGS, described in [1]). Quality control was conducted at the study level, as previously described [1, 35]; call rates were > 95% for all SNPs. Thus, 77 SNPs associated with breast cancer and their published odds ratios were used to form the PRS.

### Statistical methods

Mammographic density measures were first square root transformed and adjusted for age, 1/BMI and study, as described previously [36, 37], and residuals were used for analyses.

The 77 SNP PRS was calculated as previously described [6, 19]. Briefly, the PRS was derived for each study subject using the formula:

$$ \mathrm{PRS}={\beta}_1{x}_1+{\beta}_2{x}_2+\dots {\beta}_{\upkappa}{x}_{\upkappa}.\dots +{\beta}_n{x}_n $$

where *x*_*k*_ is the number of minor alleles (0, 1 or 2) for SNP *k*, *β*_k_ are weights, and *n* = 77 was the total number of SNPs. Under the assumption of no non-multiplicative interactions, the optimum weights *β*_k_ are the estimated per-allele log-odds ratios, and we used these weights to derive the 77 SNP PRS as previously described [6]. For missing genotypes or those excluded based on Hardy–Weinberg equilibrium *P* values < 0.001 (1.1% of the 77 genotypes), we used simple MCMC imputation to assign a probable dosage value based on the other available genotypes and risk factors [38, 39].

Pearson correlation coefficients between the continuously distributed PRS and adjusted mammographic density measures were estimated for controls separately. ORs and 95% confidence intervals (CIs) for breast cancer risk were estimated using logistic regression models and presented as the change in odds per each standard deviation of the adjusted measures (based on using controls [37]). Likelihood ratio statistics were computed to measure the strength of association of density measures, PRS, and their combinations with breast cancer risk; the baseline model for comparison was comprised of age, 1/BMI, and study. Parity, menopausal status, family history, and HT (in postmenopausal women) were also evaluated as confounders of the associations of PRS and adjusted density measures with breast cancer risk.

We estimated interactions between the adjusted mammographic density measures and the PRS and tested their significance using the likelihood ratio test (LRT). To assess the goodness of fit of a model that assumes PRS and mammographic density act multiplicatively on breast cancer risk, we performed a global Hosmer-Lemeshow goodness of fit test using deciles [40] as well as a tail-based goodness of fit test [41] to assess deviations, especially at the extremes of the risk distribution. Although primary analyses used continuous measures of density and PRS (per 1 SD), we also evaluated quintiles of PRS and quartiles of adjusted density measures to be consistent with prior studies [6, 29, 42] as well as allow for ease of interpretation, in particular for those in the lowest quartile of density. Tests of interaction and goodness of fit were performed on the subset of six population-based studies, as done in [17], given the potential for biased estimates of main effects when analyzing non-population-based studies.

Heterogeneity of association across studies was tested by including an interaction term between density measures or PRS and study, using the LRT. Statistical analyses were conducted using SAS 9.4 and R (version 3.3.1). All tests were two-sided and *P* ≤ 0.05 was considered statistically significant.

## Results

The characteristics of the 3628 cases and 5126 controls are described in Table 1 (Additional file 1: Table S2). Cases were more likely to be postmenopausal and to have a family history of breast cancer. Among postmenopausal women, cases were also more likely to have used hormone therapy (HT) (Table 1).

**Table 1:** Summary characteristics at time of mammogram and by breast case status for the eight participating studies

Characteristic	Category	Cases *N* = 3628		Controls *N* = 5126	
		*N*	%	*N*	%
Study type	Population-based or cohort	2439	67.2	3895	76.0
	Hospital-based	1189	32.8	1231	24.0
Age (years)	< 50	432	11.9	553	10.8
	50–59	1150	31.7	1462	28.5
	≥ 60	2046	56.4	3111	60.7
Parity	Nulliparous	431	12.1	582	11.5
	Parous	3141	87.9	4459	88.5
	Unknown	56		85	
Menopausal status	Pre-menopausal	540	15.0	878	17.4
	Post-menopausal	3058	85.0	4194	82.6
	Unknown	30		54	
Postmenopausal HT use in post-menopausal women	Ever	1737	59.6	2116	57.4
	Never	1179	40.4	1568	42.6
	Unknown	142		510	
BMI (kg/m^2^)	< 25	1542	42.9	2095	41.3
	≥ 25	2049	57.1	2982	58.7
	Unknown	37		49	
Family history breast cancer in first degree relatives	No	2808	81.5	3944	85.1
	Yes	637	18.5	688	14.9
	Unknown	183		494	

*HT* hormone therapy

Adjusted percent density (PD) and dense area (DA) measures were positively associated with breast cancer across all studies (Additional file 1: Table S3). For adjusted PD, there was a 1.45-fold increased risk (95% CI, 1.38–1.52) per SD of the adjusted PD (Table 2; *χ*^2^ = 156, *P* < 0.001 compared to baseline model). Further, compared to women with density in the second quartile PD, women in the top quartile had a 64% greater risk and women in the lowest quartile had a 40% lower risk of breast cancer. The associations for adjusted DA were slightly weaker than for PD, but still significant (e.g., OR 1.34 (1.28–1.41) per SD adjusted DA) (Table 2) (Additional file 1: Table S3). Associations were similar but attenuated when using population-based studies alone (Table 2) and did not materially change after adjustment for parity, menopausal status, family history, and HT (in postmenopausal women) [data not shown]. Among the population-based studies, there was some evidence for study heterogeneity (PD *p*_het_ = 0.08; DA *p*_het_ = 0.04), largely due to MMHS which had stronger associations compared to the other studies. Removal of MMHS resulted in similar associations of adjusted density measures with breast cancer (data not shown) but reduced heterogeneity (PD *p*_het_ = 0.42; DA *p*_het_ = 0.25).

**Table 2:** Associations (odds ratios, OR) for adjusted percent density (PD) and dense area (DA) measures with breast cancer risk, with or without polygenic risk score (PRS). All eight studies and restricted to cohort/population-based studies only

Adjusted density measure*	Full sample			Cohort/population-based studies only		
	*N* case/*N* control	OR (95% CI)	Adj for PRS OR (95% CI)	*N* case/*N* control	OR (95% CI)	Adj for PRS OR (95% CI)
PD (per 1 SD)	3628/5126	1.45 (1.38, 1.52)	1.42 (1.36, 1.50)	2439/3895	1.42 (1.34, 1.50)	1.40 (1.32, 1.48)
PD quartiles						
1	531/1282	0.60 (0.52, 0.70)	0.61 (0.52, 0.70)	450/1064	0.61 (0.51, 0.72)	0.62 (0.52, 0.73)
2 (Ref)	723/1281	Ref	Ref	519/984	Ref	Ref
3	946/1282	1.25 (1.09, 1.42)	1.22 (1.06, 1.39)	615/939	1.20 (1.03, 1.41)	1.18 (1.00, 1.38)
4	1428/1281	1.64 (1.44, 1.87)	1.60 (1.40, 1.82)	855/908	1.51 (1.29, 1.76)	1.48 (1.27, 1.74)
DA (per 1 SD)	3628/5126	1.34 (1.28, 1.41)	1.32 (1.26, 1.39)	2439/3895	1.36 (1.29, 1.44)	1.35 (1.28, 1.44)
DA quartiles						
1	530/1283	0.56 (0.49, 0.65)	0.57 (0.50, 0.66)	427/1040	0.58 (0.40, 0.69)	0.59 (0.50, 0.70)
2 (Ref)	764/1281	Ref	Ref	535/1003	Ref	Ref
3	964/1281	1.15 (1.01, 1.31)	1.15 (1.00, 1.31)	644/969	1.14 (0.98, 1.33)	1.13 (0.97, 1.33)
4	1370/1281	1.41 (1.23, 1.61)	1.38 (1.21, 1.59)	833/883	1.45 (1.24, 1.69)	1.44 (1.22, 1.69)

*Residuals from models adjusted for age, 1/BMI, and study

Heterogeneity of density association across population-based studies: PD *p*_het_ = 0.08; DA *p*_het_ = 0.04 (Exclusion of MMHS results in PD *p*_het_ = 0.42; DA *p*_het_ = 0.25)

*SD* standard deviation, *CI* confidence interval, *Ref* reference group, *Adj* adjusted

PRS was associated with breast cancer risk both when modeled continuously (OR = 1.52 (1.45–1.59) per SD, Table 3; *χ*^2^ = 255, *P* < 0.001 compared to baseline model), or in quintiles (Table 3). Estimates were similar when adjusted for parity, menopausal status, family history, and HT [data not shown] but slightly stronger when only including population-based studies (Table 3). There was no evidence for heterogeneity by study.

**Table 3:** Association of polygenic risk score (PRS) with breast cancer risk and evaluation of confounding due to family history and density measures (adjusted PD and adjusted DA). All eight studies combined and subset to cohort/population-based studies. All models adjusted for age, 1/BMI, and study

Model	*N* case/*N* control	PRS OR (95% CI)	+Family history OR (95% CI)	+adjusted PD OR (95% CI)	+adjusted DA OR (95% CI)
Overall sample					
Overall PRS (per 1 SD)	3628/5126	1.52 (1.45, 1.59)	1.52 (1.44, 1.59)	1.50 (1.42, 1.57)	1.50 (1.43, 1.58)
PRS quintile					
1	349/1033	0.51 (0.44, 0.60)	0.51 (0.44, 0.61)	0.53 (0.45, 0.63)	0.53 (0.45, 0.63)
2	535/1008	0.80 (0.69, 0.93)	0.78 (0.67, 0.92)	0.80 (0.69, 0.94)	0.80 (0.69, 0.94)
3 (Ref)	687/1024	1.00 (Ref)	1.00 (Ref)	1.00 (Ref)	1.00 (Ref)
4	887/1032	1.31 (1.14, 1.50)	1.31 (1.14, 1.51)	1.32 (1.14, 1.53)	1.33 (1.15, 1.53)
5	1170/1029	1.66 (1.45, 1.90)	1.65 (1.44, 1.90)	1.64 (1.43, 1.89)	1.65 (1.44, 1.91)
Cohort/population-based studies only					
Overall PRS (per 1 SD)	2439/3895	1.56 (1.48, 1.66)	1.55 (1.47, 1.65)	1.54 (1.45, 1.63)	1.55 (1.46, 1.64)
PRS quintile					
1	232/786	0.51 (0.42, 0.62)	0.51 (0.42, 0.62)	0.53 (0.43, 0.65)	0.53 (0.43, 0.64)
2	361/776	0.80 (0.66, 0.95)	0.79 (0.66, 0.94)	0.80 (0.67, 0.96)	0.80 (0.67, 0.96)
3 (Ref)	464/789	1.00 (Ref)	1.00 (Ref)	1.00 (Ref)	1.00 (Ref)
4	589/793	1.30 (1.10, 1.53)	1.28 (1.08, 1.52)	1.29 (1.09, 1.54)	1.30 (1.10, 1.54)
5	793/751	1.79 (1.52, 2.11)	1.76 (1.50, 2.08)	1.76 (1.49, 2.08)	1.77 (1.50, 2.09)

Heterogeneity of PRS association by study: *P* = 0.84 for population based studies

PRS quintiles: quintile 1, − 1.411 to − 0.014; quintile 2, − 0.015 to 0.280; quintile 3, 0.281 to 0.542; quintile 4, 0.543 to 0.885; quintile 5, 0.886 to 2.583

*SD* standard deviation, *CI* confidence interval, *Ref r*eference group, *Adj* adjusted

PRS and adjusted density measures were only weakly correlated (Pearson correlation 0.06, *P* < 0.001 for adjusted PD and 0.05, *P* < 0.001 for adjusted DA using controls). Adjusting for PRS made little change to the association between adjusted density measures and breast cancer risk (e.g., OR per 1 SD for adjusted PD, 1.42, 95% CI 1.36–1.50; Table 2). Similarly, adjustment for density measures had very little impact on the association between PRS and risk (Table 3).

### Interactions between adjusted density measures and PRS on breast cancer risk

**Fig. 1:**
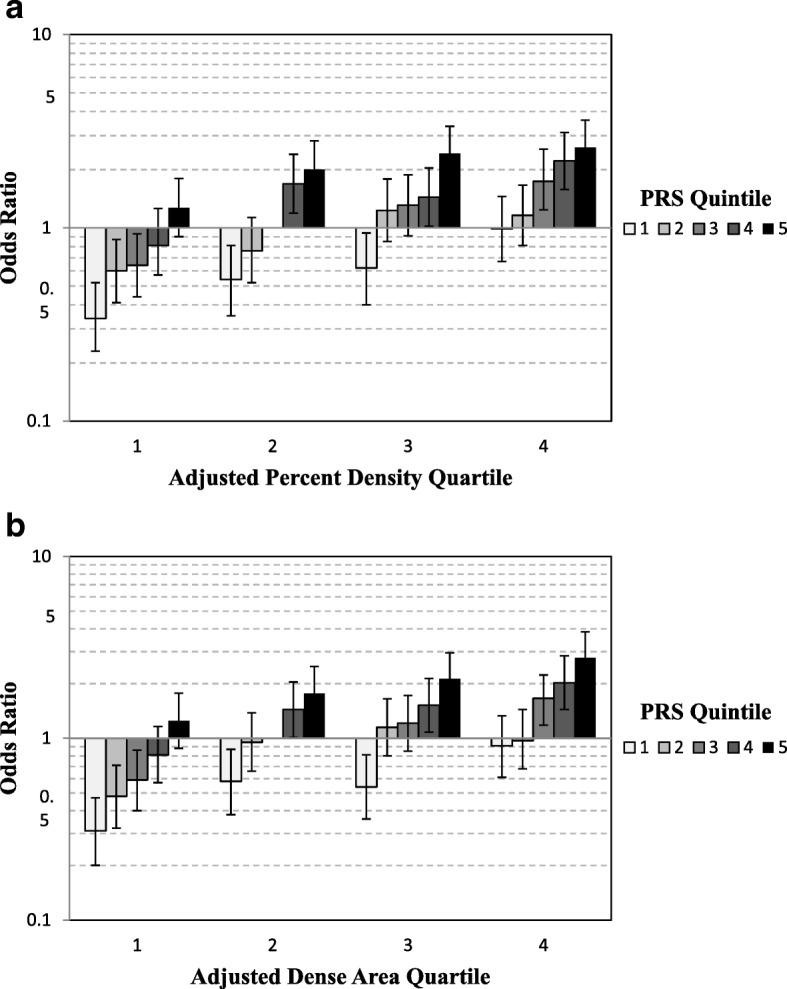
Joint association of quartiles of adjusted density phenotypes and quintiles PRS with breast cancer risk, adjusted for age, 1/BMI, and study. Quartiles adjusted percent density and PRS quintile with breast cancer risk (**a**). Quartiles of adjusted dense area and PRS quintile with breast cancer risk (**b**). PRS quintiles: quintile 1, − 1.411 to − 0.014; quintile 2, − 0.015 to 0.280; quintile 3, 0.281 to 0.542; quintile 4, 0.543 to 0.885; quintile 5, 0.886 to 2.583. Reference category is PRS quintile 3 and density quartile 2

**Fig. 2:**
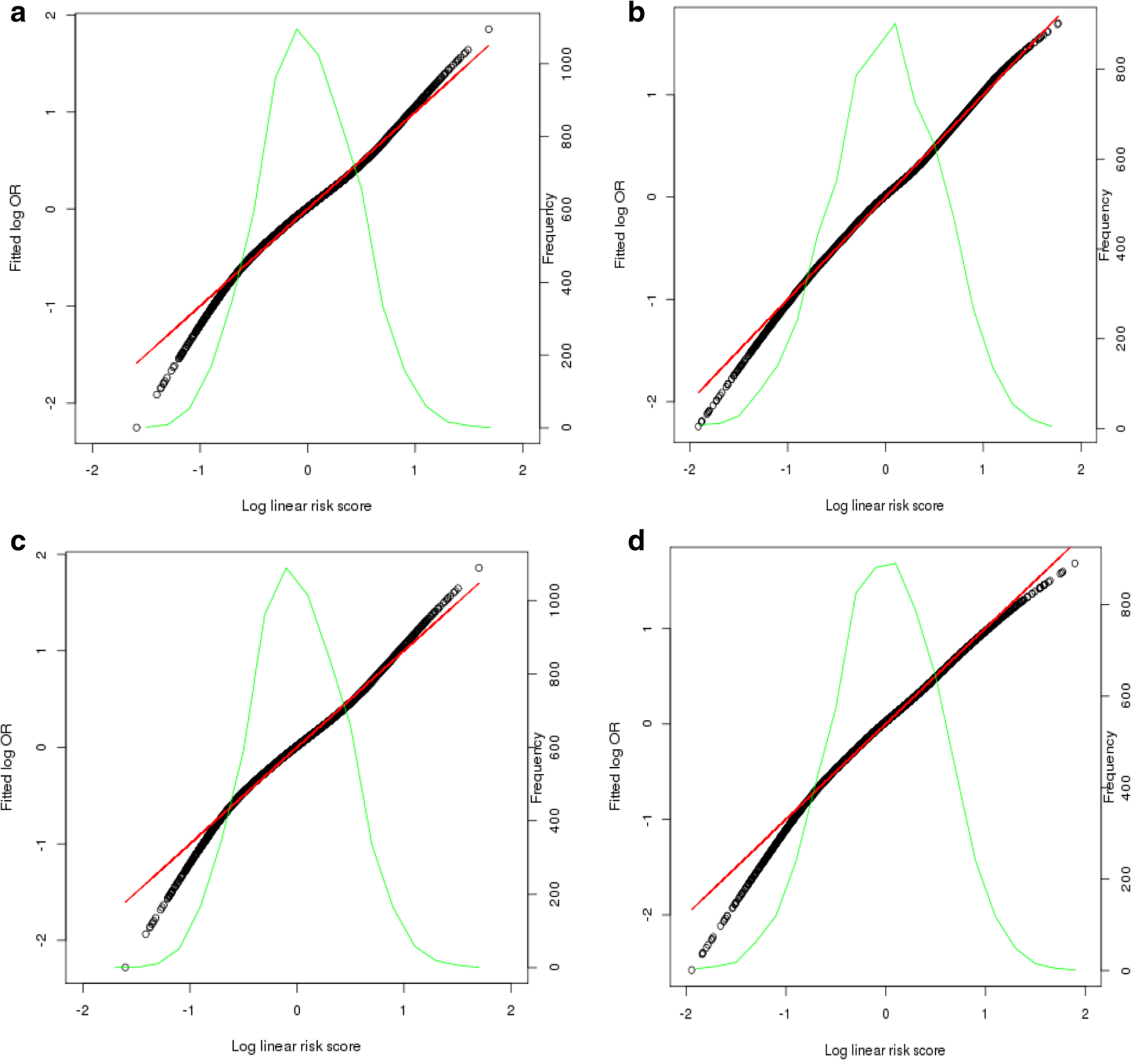
Tail-based test results from models with continuous adjusted density measures and PRS on breast cancer risk. Population-based studies. Models of adjusted percent density and PRS without interaction (**a**) and with multiplicative interaction included (**b**). Models with adjusted dense area and PRS without interaction (**c**) and with multiplicative interaction included (**d**)

Among the population-based studies, there was no evidence of an interaction between PRS and adjusted PD, whether assessed as continuous (per 1 SD) or categorical (quartiles PD/quintiles PRS) variables; this included evaluation by likelihood ratio tests [[OR_int_ (95% CI) = 0.96 (0.91,1.02), *χ*^2^_LRT_ = 1.6_,_ *P*_LRT_ = 0.21 for continuous and *P* = 0.42 for categorical] (Fig. 1) and global (*P* > 0.09) or tail-based (*P* > 0.23) goodness of fit tests (Fig. 2; Additional file 1: Table S4). Findings were generally similar for adjusted DA and PRS on breast cancer (Additional file 1: Table S4) (Figs. 1 and 2). Results were unchanged when excluding MMHS.

The multiplicative association can be seen in the similarities of the risk estimates from the joint association of PRS and density measures to the risk expected from the individual main effect models (Tables 2 and 3; Fig. 1). The joint association (OR) was 2.60 in the highest categories of adjusted PD and PRS and 0.34 in the lowest categories relative to the reference category (Fig. 1). These estimates are very similar to the predicted relative risk estimates resulting from multiplying the individual OR associated with each category of PRS and adjusted PD from the main effect models [i.e., OR(fourth quartile PD) × OR(fifth quintile PRS) = 2.70; OR(first quartile PD) × OR(first quintile PRS) = 0.31].

## Discussion

This is the first study to have examined the joint association of a 77-SNP PRS and continuous mammographic density measures on breast cancer risk. We found that the combined associations of the PRS and adjusted density measures on breast cancer are well described by a multiplicative model. These results imply that either adjusted percent density or absolute dense area measures can be incorporated as continuous measures into risk models with PRS in a straightforward manner, without interaction terms. Moreover, the two measures are close to uncorrelated; as a result, the risk discrimination afforded by using both measures is much greater than using either alone.

Our findings are consistent with those from the few prior studies evaluating PRS and density measures. Two studies evaluated the contribution of the PRS to the Breast Cancer Surveillance Consortium or BCSC model, which includes the BI-RADS four category density measure [11, 19]. Both studies found significant improvement in the discrimination with the PRS. One of these [19] evaluated an interaction between the BI-RADS density measure and the PRS, finding no departure from a multiplicative model and little correlation between the BI-RADS density and PRS measures. van Veen et al. [43] recently examined an 18-SNP PRS, IBIS 10-year risk, and a visual measure of mammographic density (adjusted for BMI, age) with breast cancer risk among women in a mammography screening practice. Assuming independence between the PRS, IBIS model, and mammographic density, they found that the PRS added substantial information to a model with IBIS risk and mammographic density. They also found only a weak correlation between the PRS, adjusted density, and the IBIS risk model estimate, consistent with our results and those of Vachon et al. [19] Similarly, using data from the Nurses’ Health Studies, Zhang et al. [16] found significant improvement in discrimination when a 67-SNP PRS was added to either the BCRAT model or the Rosner-Colditz model. Addition of a continuous measure of mammographic density also significantly improved the discrimination of both models. However, this paper did not specifically evaluate any interaction between mammographic density and the PRS. Our paper is the first to examine the joint association of continuous density measures with a PRS.

Our findings are consistent with joint effects of PRS with other breast cancer risk factors. Two studies have examined the joint association of a PRS with reproductive variables (age at menarche, parity, age at first birth), alcohol intake, postmenopausal hormone therapy, and BMI on breast cancer. The first involved a 77-SNP PRS examined in up to 28,241 cases and 30,445 controls in BCAC, finding that most associations were consistent with a multiplicative association [17]. An earlier study examined a 24-SNP PRS with the same risk factors, using 17,171 cases and 19,862 controls from the Breast and Prostate Cancer Cohort Consortium (BPC3) and also did not find deviation from the multiplicative model [18].

Our study used quantitative density measures assessed from digitized film mammograms, primarily using the Cumulus software. Although this measure allowed us to evaluate a more precise density measure than the four BI-RADS categories evaluated previously, it is not used in clinical practice as it is not fully automated. Some centers now use commercially available automated density measures such as Volpara which provide a continuous volumetric density in addition to a BI-RADS-like categorical estimate. We and others have shown that these automated measures have a similar ability to predict risk as the BI-RADS clinical density measure and the area-based measures used in the current report [42, 44]. It is likely that our results on the combined effect of the SNP and thresholding density measures presented here will translate to the automated volumetric measures, but this still needs to be evaluated directly.

Since the 77-SNP PRS was established and validated, additional common genetic variants have been identified for breast cancer risk, and these will allow a more informative PRS to be developed [2–4, 35]. The 77 SNPs contributing to the PRS used in this report are estimated to explain 14% of the familial risk, and additional SNPs identified are estimated to explain another 4% [2]. Of the 77 SNPs, nine have been shown to be associated with mammographic density phenotypes but together they explain less than 1.5% of the between-woman variation in these adjusted density traits [31]. Despite this overlap, the PRS is almost uncorrelated with the density measures, and adjustment for PRS resulted in minimal changes in the association between adjusted density measures and breast cancer and vice versa.

Some SNPs have been identified to be more strongly associated with ER-positive or ER-negative disease [2, 4, 45, 46]. The majority of the 77 SNPs in the current PRS are associated with ER-positive breast cancer, with only 27 associated at *P* < 10^− 4^ with ER-negative breast cancer [1, 2, 6]. Mammographic density has been shown to be a risk factor for both ER-positive and ER-negative breast cancer and all 9 of the SNPs associated with density are associated with both subtypes [1, 2, 47–49]. Future analyses which consider the joint associations of the subtype-specific PRS and mammographic density with breast cancer risk will be worthwhile.

Important strengths of this analysis include the largest dataset to date to examine the combined associations of PRS and mammographic density on breast cancer risk, the use of quantitative density measures that were standardized across studies, and a common genotyping platform with standard quality control procedures for the majority of studies. We recognize, however, that our results are strictly generalizable to women of European ancestry only. We also note some overlap between our studies with those used to identify the associated SNPs and develop the PRS. However, while this could have led to some overfitting and hence overestimation of the risk gradient for the PRS, this would not have affected the primary conclusion that associations of the PRS and density are almost completely independent and not confounded. Also, two of the largest cohort studies in our sample (MMHS, NHS) consisting of over 1300 cases were not included in the studies used for development of the 77-SNP PRS and estimation of the PRS risk gradients (1.7 and 1.4 per unit SD for PD) were similar to those estimated from the remaining studies. Finally, as noted above, additional work will be necessary to confirm our findings with an updated PRS and novel mammographic density measures [50, 51] as they become available.

## Conclusion

In summary, we confirm continuous mammographic density measures and PRS are two of the strongest risk factors for breast cancer and can be included in risk models without interaction terms. Absolute risk associated with higher density will be larger for women at high SNP-based inherited risk.

## Additional file

Additional file 1: **Table S1.** Study design and characteristics for eight participating studies. **Table S2.** Characteristics of cases and non-cases by study (% of non-missing). **Table S3.** Associations (OR, 95% CI) of adjusted percent density (PD), adjusted dense area (DA) and polygenic risk score (PRS) with breast cancer by study. Adjusted for age and BMI. **Table S4.** Goodness of fit test *P* values for joint effect of adjusted* density measures with 77-SNP polygenic risk score (PRS) on breast cancer, based on six population-based studies. PRS and density measures are continuous (per 1 standard deviation, SD). (DOCX 34 kb)

## Abbreviations

BCAC: Breast Cancer Association Consortium
BCRAT: Breast Cancer Risk Assessment Tool
BCSC: Breast Cancer Surveillance Consortium
BI-RADS: Breast Imaging Reporting and Data System
BMI: Body mass index
BPC3: Breast and Prostate Cancer Cohort Consortium
CC: Craniocaudal
CI: Confidence interval
DA: Dense area
GWAS: Genome-wide association study
HT: Hormone therapy
IBIS: International Breast Cancer Intervention Study
LR: Likelihood-ratio
MCMC: Markov Chain Monte Carlo
MLO: Mediolateral oblique
OR: Odds ratio
PD: Percent density
PRS: Polygenic risk score
SD: Standard deviation
SNP: Single nucleotide polymorphism
