# Supplementary material for: Joint association of mammographic density adjusted for age and body mass index and polygenic risk score with breast cancer risk
Source: Breast Cancer Res. 2019 May 22;21:68. doi: 10.1186/s13058-019-1138-8 (PMC6532188; doi:10.1186/s13058-019-1138-8)
Supplement: Supplementary file 1 — Table S1. Study design and characteristics for eight participating studies. Table S2. Characteristics of cases and non-cases by study (% of non-missing). Table S3. Associations (OR, 95% CI) of adjusted percent density (PD), adjusted dense area (DA) and polygenic risk score (PRS) with breast cancer by study. Adjusted for age and BMI. Table S4. Goodness of fit test P values for joint effect of adjusted* density measures with 77-SNP polygenic risk score (PRS) on breast cancer, based on six population-based studies. PRS and density measures are continuous (per 1 standard deviation, SD). (DOCX 34 kb) [file 13058_2019_1138_MOESM1_ESM.docx]

| **Online Table 1.** Study design and characteristics for eight participating studies | | | | | | | | | | |
| --- | --- | --- | --- | --- | --- | --- | --- | --- | --- | --- |
| **Study name, acronym** | **Design** | **Number**  **of Cases**  **/Controls** | **Source of covariate data** | | | **Density software** | **Mammogram Film view** | **Breast side** | | **Genotyping**  **(GWAS or iCOGS)** |
|  |  |  | Repro-  ductive  variables | Anthropometric variables | Time between mammogram and data collection |  |  | Cases | Controls |  |
| BBCC | CC | 512/367 | Quest. | Self-report | Within 30 days | Madena | CC | Average | Average | iCOGS |
| EPIC* | Cohort | 86/968 | Quest | Measured | 3 years prior | Cumulus | MLO | Contra | Average | iCOGS |
| MCBCS | CC | 677/864 | Quest | Measured | Within 30 days | Cumulus | CC | Contra | L | iCOGS |
| MCCS* | Nested CC | 68/28 | Quest | Measured | 3 years prior | Cumulus | CC | R | R | iCOGS |
| MEC* | Nested CC | 110/101 | Quest | Self-report | Within 5 years prior | Cumulus | CC | Average | Average | iCOGS |
| MMHS* | Nested CC | 456/1166 | Self | Measured | Within 30 days | Cumulus | CC | Average | Average | iCOGS |
| NHS* | Nested CC | 850/849 | Quest | Self-report | Within 2 years | Cumulus | CC | Average | Average | GWAS |
| SASBAC* | CC | 869/783 | Quest | Self-report | Mean 1 year post | Cumulus | MLO | Contra | L/R | iCOGS |
| *Population-based studies. | | | | | | | | | | |

| **Online Table 2.** Characteristics of cases and non-cases by study (% of non-missing). | | | | | | | | | | | | | | | | |
| --- | --- | --- | --- | --- | --- | --- | --- | --- | --- | --- | --- | --- | --- | --- | --- | --- |
| **Characteristic** | **BBCC** | | **EPIC** | | **MCBCS** | | **MCCS** | | **MEC** | | **MMHS** | | **NHS** | | **SASBAC** | |
|  | **Cases**  **N=512** | **Non-cases**  **N=367** | **Cases**  **N=86** | **Non-cases**  **N=968** | **Cases**  **N=677** | **Non-cases**  **N=864** | **Cases**  **N=68** | **Non-cases**  **N=28** | **Cases**  **N=110** | **Non-cases**  **N=101** | **Cases**  **N=456** | **Non-cases**  **N=1166** | **Cases**  **N=850** | **Non-cases**  **N=849** | **Cases**  **N=869** | **Non-cases**  **N=783** |
|  | **%** | **%** | **%** | **%** | **%** | **%** | **%** | **%** | **%** | **%** | **%** | **%** | **%** | **%** | **%** | **%** |
| Age <50 | 17 | 25 | 0 | 0 | 27 | 17 | 7 | 11 | 11 | 27 | 18 | 20 | 6 | 6 | 1 | 1 |
| Age 50-59 | 26 | 31 | 19 | 17 | 30 | 28 | 44 | 46 | 35 | 38 | 25 | 27 | 37 | 38 | 34 | 32 |
| Age ≥ 60 | 57 | 44 | 81 | 83 | 43 | 55 | 49 | 43 | 55 | 36 | 57 | 53 | 57 | 56 | 65 | 67 |
| Parous | 84 | 82 | 84 | 87 | 88 | 85 | 85 | 93 | 84 | 79 | 88 | 89 | 92 | 94 | 87 | 92 |
| Post-menopausal | 80 | 79 | 78 | 76 | 72 | 81 | 78 | 89 | 92 | 72 | 75 | 75 | 92 | 91 | 97 | 98 |
| Ever HRT (Post meno) | 39 | 55 | 33 | 37 | 64 | 74 | 22 | 24 | 80 | 82 | 67 | 66 | 74 | 72 | 52 | 43 |
| BMI ≥ 25 | 55 | 44 | 67 | 65 | 64 | 67 | 63 | 57 | 44 | 46 | 70 | 65 | 51 | 49 | 51 | 53 |
| Family Hx BC | -- | -- | 12 | 8 | 23 | 19 | -- | -- | 10 | 6 | 31 | 21 | 16 | 12 | 15 | 9 |

| **Online Table 3.** Associations (OR, 95% CI) of adjusted percent density (PD), adjusted dense area (DA) and polygenic risk score (PRS) with breast cancer by study. Adjusted for age and BMI. | | | | | | | | |
| --- | --- | --- | --- | --- | --- | --- | --- | --- |
| **Model** | **BBCC**  **(512/367)**  **OR (95% CI)** | **EPIC***  **(86/968)**  **OR (95% CI)** | **MCBCS**  **(677/864)**  **OR (95% CI)** | **MCCS***  **(68/28)**  **OR (95% CI)** | **MEC***  **(110/101)**  **OR (95% CI)** | **MMHS* (456/1166)**  **OR (95% CI)** | **NHS***  **(850/849)**  **OR (95% CI)** | **SASBAC***  **(869/783)**  **OR (95% CI)** |
| PD (per SD) | 1.2 (1.0, 1.4) | 1.3 (1.0, 1.7) | 1.9 (1.7, 2.2) | 1.6 (1.0, 2.6) | 1.5 (1.2, 1.9) | 1.7 (1.5, 1.9) | 1.4 (1.3, 1.6) | 1.3 (1.2, 1.4) |
| PD Quartiles  1  2 (ref)  3  4 | 1.4 (0.4, 5.1)  ref  1.0 (0.6, 1.8)  1.6 (1.0, 2.7) | 0.9 (0.5, 1.9)  ref  1.4 (0.7, 2.5)  1.8 (1.0, 3.3) | 0.5 (0.4, 0.7)  ref  1.5 (1.1, 1.9)  2.4 (1.8, 3.2) | 0.8 (0.3, 2.7)  ref  2.0 (0.5, 8.4)  3.7 (0.8, 17.9) | 0.5 (0.2, 1.3)  ref  1.4 (0.5, 4.1)  1.7 (0.7, 3.8) | 0.4 (0.3, 0.6)  ref  1.2 (0.9, 1.6)  1.7 (1.3, 2.3) | 0.7 (0.5, 1.0)  ref  1.4 (1.0, 1.9)  1.9 (1.4, 2.6) | 0.6 (0.4, 0.8)  ref  1.1 (0.8, 1.4)  1.0 (0.7, 1.3) |
| DA (per SD) | 0.9 (0.8, 1.1) | 1.3 (1.0, 1.6) | 1.7 (1.5, 2.0) | 1.7 (1.0, 3.0) | 1.7 (1.3, 2.3) | 1.6 (1.4, 1.9) | 1.3 (1.2, 1.4) | 1.3 (1.1, 1.4) |
| DA Quartiles  1  2 (ref)  3  4 | 1.2 (0.2, 7.3)  ref  0.5 (0.2, 1.1)  0.5 (0.2, 1.1) | 1.1 (0.6, 2.2)  ref  0.9 (0.5, 1.8)  1.6 (0.9, 2.8) | 0.5 (0.4, 0.7)  ref  1.3 (1.0, 1.7)  1.7 (1.3, 2.4) | 0.9 (0.3, 2.7)  ref  1.6 (0.4, 6.4)  3.8 (0.6, 22.1) | 0.4 (0.2, 1.0)  ref  1.3 (0.5, 3.1)  1.9 (0.8, 4.2) | 0.5 (0.4, 0.7)  ref  1.3 (1.0, 1.7)  1.5 (1.1, 2.2) | 0.6 (0.4, 0.8)  ref  1.1 (0.8, 1.5)  1.7 (1.3, 2.3) | 0.6 (0.4, 0.8)  ref  1.1 (0.8, 1.5)  1.0 (0.7, 1.3) |
| PRS (per SD) | 1.3 (1.1, 1.5) | 1.4 (1.1, 1.8) | 1.5 (1.3, 1.6) | 1.2 (0.7, 2.0) | 1.6 (1.2, 2.1) | 1.6 (1.4, 1.8) | 1.6 (1.4, 1.8) | 1.6 (1.4, 1.7) |
| PRS Quintiles  1  2  3 (ref)  4  5 | 0.8 (0.5, 1.3)  0.8 (0.5, 1.3)  Ref  1.2 (0.8, 1.8)  1.5 (1.0, 2.3) | 0.6 (0.3, 1.3)  0.4 (0.2, 0.9)  Ref  1.0 (0.5, 1.9)  1.1 (0.6, 2.1) | 0.4 (0.3, 0.6)  0.8 (0.5, 1.1)  Ref  1.4 (1.0, 1.9)  1.3 (0.9, 1.7) | 0.6 (0.1, 2.6)  2.8 (0.6, 13.4)  Ref  1.9 (0.5, 7.7)  1.5 (0.4, 5.3) | 0.3 (0.1, 0.9)  0.7 (0.2, 2.0)  Ref  1.2 (0.5, 3.1)  1.4 (0.6, 3.3) | 0.5 (0.3, 0.5)  0.7 (0.5, 1.0)  Ref  1.3 (0.9, 1.8)  1.7 (1.2, 2.4) | 0.6 (0.4, 0.8)  0.8 (0.6, 1.1)  Ref  1.3 (1.0, 1.8)  2.1 (1.6, 2.8) | 0.4 (0.3, 0.6)  0.9 (0.6, 1.2)  Ref  1.3 (0.9, 1.7)  1.8 (1.3, 2.4) |
| *Population-based studies used for analyses of joint associations. | | | | | | | | |

| **Online Table 4.** Goodness of fit test p-values for joint effect of adjusted* density measures with 77-SNP polygenic risk score (PRS) on breast cancer, based on six population-based studies. PRS and density measures are continuous (per 1 standard deviation, SD) | | | | | | |  |
| --- | --- | --- | --- | --- | --- | --- | --- |
| **Variables included in models with 77-SNP PRS (per SD)** | **N studies**** | **N Cases/controls** | **Tail-based**  **goodness of fit test** | | **Hosmer and Lemeshow**  **goodness of fit test** | |  |
|  |  |  | No interaction | With interaction | No interaction | With interaction | |
| Adjusted* percent density (per SD) | 6 | 2439/3895 | 0.23 | 0.70 | 0.09 | 0.09 | |
| Adjusted* dense area (per SD) | 6 | 2439/3895 | 0.44 | 0.88 | 0.27 | 0.39 | |
| *Residuals from model adjusted for age, 1/BMI and study  **Population-based studies include MEC, MMHS, MCCS, NHS, EPIC, SASBAC | | | | | | | |
